# Lung Volumes in a Mouse Model of Pulmonary Allergic Inflammation

**DOI:** 10.1007/s00408-024-00730-1

**Published:** 2024-07-17

**Authors:** Andrés Rojas-Ruiz, Magali Boucher, Cyndi Henry, Rosalie Packwood, Jorge Soliz, Ynuk Bossé

**Affiliations:** Institut Universitaire de Cardiologie et de Pneumologie de Québec (IUCPQ), Université Laval, Pavillon M, Room 2687, 2725, Chemin Sainte-Foy, Québec, QC G1V 4G5 Canada

**Keywords:** Air trapping, Lung mechanics, Mouse models, Residual volume, Total lung capacity

## Abstract

**Purpose:**

Air trapping, often attested in humans by elevated residual volume (RV) and ratio of RV on total lung capacity (RV/TLC), is frequently observed in asthma. Confirming these alterations in experimental asthma would be important for translational purposes. Herein, lung volumes were investigated in a mouse model of pulmonary allergic inflammation.

**Methods:**

Eight- to 10-week-old male C57BL/6 and BALB/c mice were exposed once daily to intranasal house dust mite (HDM) for 10 consecutive days. All readouts were measured 24 h after the last exposure. Lung volumes were assessed with the flexiVent using a new automated method consisting of degassing the lungs followed by a full-range pressure–volume maneuver. The weight and the volume of the lungs were also measured *ex vivo* and a lobe was further processed for histological analyses.

**Results:**

HDM exposure led to tissue infiltration with inflammatory cells, goblet cell hyperplasia, thickening of the airway epithelium, and elevated *ex vivo* lung weight and volume. It also decreased TLC and vital capacity but without affecting RV and RV/TLC. These observations were similar between the two mouse strains.

**Conclusion:**

Alterations of lung volumes in a murine model of pulmonary allergic inflammation are inconsistent with observations made in human asthma. These discrepancies reflect the different means whereby lung volumes are measured between species. The invasive method used herein enables RV to be measured more precisely and without the confounding effect of air trapping, suggesting that changes in RV and RV/TLC using this method in mice should be interpreted differently than in humans.

## Introduction

Lung volumes in humans are routinely measured in laboratories of respiratory physiology. They are typically measured by whole-body plethysmography. The test requires a series of breathing maneuvers, including a bout of panting against a close shutter [1, 2]. By monitoring the pressure inside the body box and the subject’s mouth during the panting (which is the same as the pressure inside the lungs when the shutter is closed), the amount of air in the lungs at the end of a normal expiration can be deduced by the law of Boyle–Mariotte [1, 2]. This volume is actually a capacity (a volume being a distinct fraction of the whole lung volume and a capacity being a sum of these distinct volumes), called functional residual capacity (FRC). Together with full inspiratory and expiratory breaths inside the body box, FRC can be used to deduce three other capacities, namely inspiratory capacity (IC), forced vital capacity (FVC), and total lung capacity (TLC), as well as four volumes, namely residual volume (RV), expiratory reserve volume (ERV), tidal volume (TV), and inspiratory reserve volume (IRV). Lung volumes provide important insights regarding the root cause of respiratory symptoms and are useful for diagnosing respiratory diseases [2]. In asthma, the two most affected of these volumes and capacities are RV and FRC [3–5]. Both are typically elevated in asthma and, in conjunction with an elevated RV/TLC ratio, they are interpreted as signs of air trapping.

In comparison to humans, measuring lung volumes in mice poses additional challenges, including their small size and their conspicuous lack of cooperativity. Robichaud and co-workers have recently developed a fully automated method to assess lung volumes in rodents [6]. The method relies on pressure–volume (*P*–*V*) maneuvers that were initially developed to study lung diseases affecting the parenchyma, such as chronic obstructive pulmonary disease and fibrosing lung diseases [7–9]. *P*–*V* maneuvers are not typically utilized in mouse models of asthma, probably because asthma is not considered a parenchymal disease. Yet, some readouts deduced from the *P*–*V* loop, such as lung compliance, were showed to be altered in subgroups of asthmatic patients [10, 11]. Hysteresis, which is the area within the *P*–*V* loop, may also be altered in asthma given that hysteresis is greatly influenced by smooth muscle tone [12] and that smooth muscle tone is elevated in asthma [13]. Therefore, important complementary insights may be gained by investigating the *P*–*V* loop in a mouse model of asthma. The goal of the present study was to use the new automated method by Robichaud and co-workers [6] to determine whether lung volumes, as well as other readouts from the *P*–*V* loop, such as hysteresis and compliance, are affected in a mouse model of pulmonary allergic inflammation.

## Methods

### Mice

Twenty-four male BALB/c mice (Charles River, Saint-Constant, Canada) and 24 male C57BL/6 mice (Jackson, Bar Harbor, MA, USA) were studied at 8–10 weeks of age. Since lung mechanics differ significantly between BALB/c and C57BL/6 mice [14], we reasoned that measuring and comparing the lung volume response to HDM in these two strains may provide additional insights about the underlying mechanisms. All methods were approved by the Committee of Animal Care of *Université Laval* following the guidelines from the Canadian Council on Animal Care (2020-652-4) and complied with the ARRIVE guidelines**.**

### Model of Pulmonary Allergic Inflammation

**Fig. 1:**
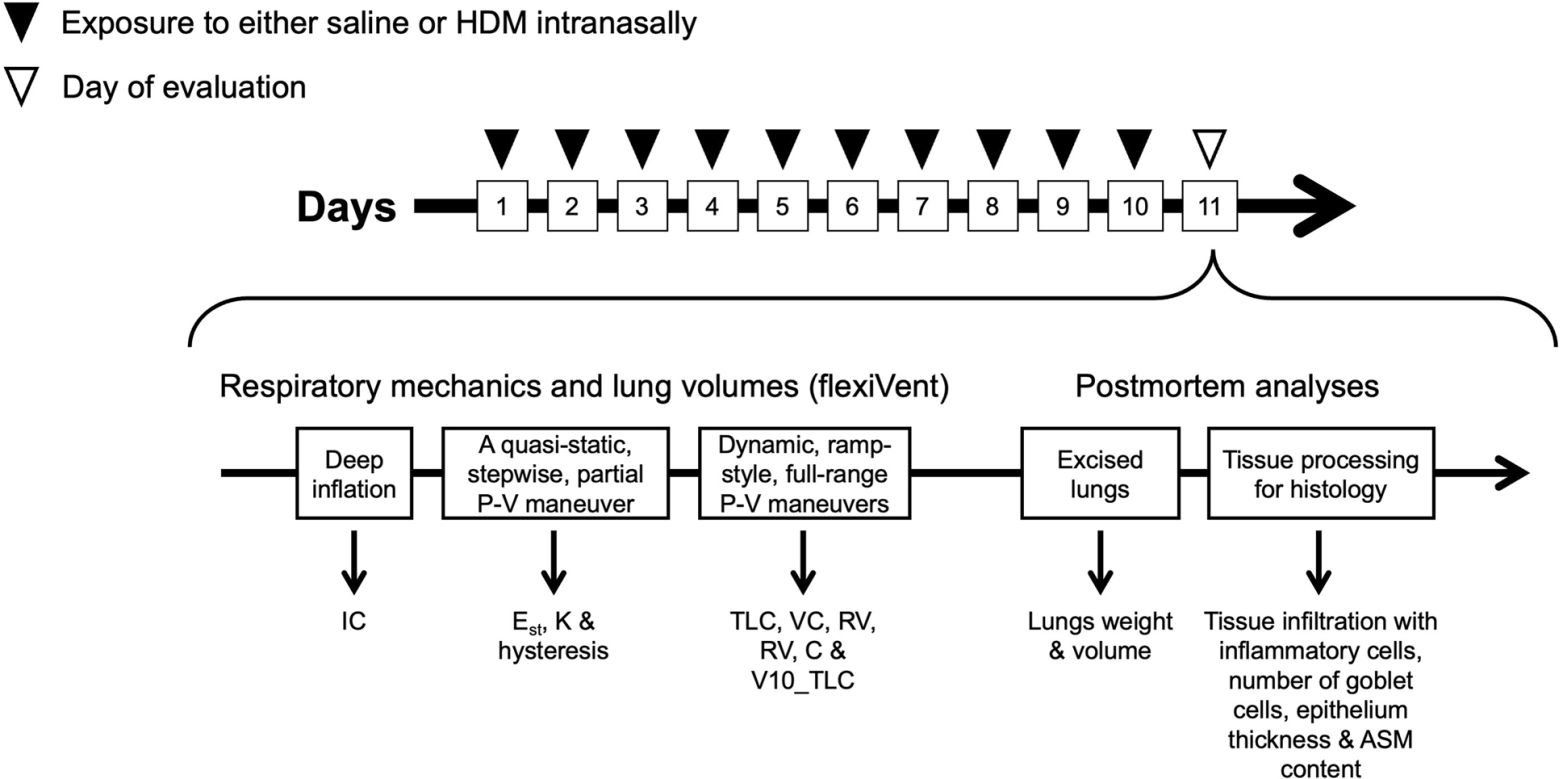
Schematic depicting the sequence of interventions. See methods for further details. Abbreviations: ASM, airway smooth muscle; C, respiratory system compliance; *E*_st_, quasi-static elastance of the respiratory system; HDM, house dust mite; IC, inspiratory capacity; K, the parameter K of Salazar–Knowles equation (a volume-independent indicator of the tissue compliance of the respiratory system); *P*–*V*, pressure–volume; RV, residual volume; TLC, total lung capacity; VC, vital capacity; V10_TLC, lung volume at 10 cm H_2_O expressed in percentage of TLC

Pulmonary allergic inflammation was induced as previously described [15–17]. Briefly, mice were exposed to either 25 μL of saline or 25 μL of 2 mg/mL of house dust mite (HDM) extract (*D. pteronyssinus, lot number* 360923; Greer, Lenoir, NC) diluted in saline to induce pulmonary allergic inflammation (Fig. 1). The endotoxin concentration was 47.3 EU per mg of HDM extract. The exposure occurred once daily for 10 consecutive days under isoflurane anesthesia via an intranasal instillation. All measurements were made 24 h after the last exposure.

### Lung Volumes

Mice were first anesthetized and put under general analgesia using ketamine (100 mg/kg) and xylazine (10 mg/kg). Their body weight was then measured and they were then tracheotomized, connected to the flexiVent (FX Module 2, SCIREQ, Montreal, QC, Canada) through an 18-gauge cannula in a supine position, and then ventilated as previously described [14]. Lung volumes were also measured by the flexiVent using three distinct maneuvers [6, 14] (Fig. 1). The maximal pressure in all three maneuvers was set to 40-cm H_2_O. All pressures reported herein refer to trans-respiratory pressure. The pressure was pushed beyond 25-cm H_2_O [18], because the effect of fluid engorgement within the lungs was shown to be more perceptible at higher pressures [19].

The first maneuver, called the deep inflation, consists of inflating the lungs from 3- to 40-cm H_2_O in 3 s and then maintaining that pressure for another 3 s. The volume that enters the lungs from the beginning to the end of the maneuver is considered the inspiratory capacity (*IC*).

The second one, called the quasi-static, stepwise, pressure-controlled partial pressure–volume (*P*–*V*) maneuver (hereinafter called the partial *P*–*V* maneuver), consists of sequentially inflating the lungs through eight steps of increasing pressure (3- to 40-cm H_2_O) and then deflating it through eight steps of decreasing pressure (40- to 3-cm H_2_O). The entire maneuver lasts 16 s. Volume changes at the different holding pressures are recorded and then plotted to form the inflation and deflation limbs of the *P*–*V* loop. The descending limb of the *P*–*V* loop is then fitted to the Salazar–Knowles Eq. [20] to deduce the parameter K, which is a volume-independent indicator of tissue compliance of the respiratory system [21]. Hysteresis, which is the area within the *P*–*V* loop, and the quasi-static elastance (*E*_st_) was also extracted from the *P*–*V* loop [21]. *E*_st_ was calculated from the inverse of the slope of the fit of the Salazar–Knowles equation at 5 cm H_2_O.

The third one, called the dynamic, ramp-style, full-range *P*–*V* maneuver (hereinafter called the full-range *P*–*V* maneuver) was performed as previously described [22] with a modification [19]. It starts by degassing the lungs by ventilating with 100% oxygen for 5 min. The ventilation includes a deep inflation every 1 min to help recruit the trapped air behind closed airways, thereby ensuring a complete substitution of air for oxygen. The ventilation is then stopped for 5 min. During this period of apnea, all the gas in the lungs, which is now made of 100% oxygen can be absorbed, which would have not been the case with air that contains non-absorbable gases, such as nitrogen. The lungs consequently deflated until the alveoli and small airways collapse to achieve a lung volume near zero. A slow constant inflow of air (5 mL/min) is then reintroduced in degassed lungs to inflate them until the pressure reaches 40-cm H_2_O. The flow is then reversed to deflate the lungs at the same rate until −10 cm H_2_O is reached. The pressure excursion from −10- to 40-cm H_2_O is then repeated twice for quality control purposes. Once completed, three P–V loops can be visualized: one starting from zero volume at zero pressure and two starting from −10-cm H_2_O. The volume entering from degassed lungs at zero-cm H_2_O to 40-cm H_2_O is considered the total lung capacity (TLC). The difference in volume between degassed lungs and the volume at −10-cm H_2_O at the end of the deflating limb is considered the residual volume (RV). The difference between TLC and RV is then the vital capacity (VC).

Two additional readouts are measured from the full-range *P*–*V* maneuver, namely C and V_10__TLC. C represents the compliance of the respiratory system, calculated from the slope of the linear part of the deflation limb between 3- and 8-cm H_2_O. V_10__TLC is the volume of the lungs at 10-cm H_2_O expressed in percentage of TLC. It is used to describe the shape of the deflation limb and, similar to the K in Salazar–Knowles equation, is considered a volume-independent indicator of compliance of the respiratory system [9, 21].

### Euthanasia

The full-range P–V maneuver is terminal. Mice died during the apneic period. Exsanguination was still performed at the end of the measurement to ascertain death.

### Wet Weight and Physical Lung Volume

The lungs were surgically removed, cleaned, and weighed. To measure their volume, they were immerged into a milliliter-graduated cylinder filled with Krebs solution and the change in volume displaced by the lungs was recorded.

### Histology

Histology was performed on the left lung in half of mice (*n* = 6). Histologic alterations seen in this specific model of pulmonary allergic inflammation were previously characterized [14, 15, 17]. It was repeated herein to confirm the establishment of pulmonary allergic inflammation. Lung sections stained with hematoxylin and eosin (H&E) were used to quantify tissue infiltration with inflammatory cells. Sixteen non-overlapping photomicrographs (1440 × 904 pixels) from 4 non-contiguous lung sections were blindly scored from zero (no inflammation) to 5 (very severe inflammation). Lung sections stained with Periodic acid–Schiff (PAS) with alcian blue were used to count the number of goblet cells. All airways cut transversally in 4 non-contiguous lung sections were analyzed, representing 14–33 airways per mouse (average of 24.2 ± 5.4). Lung sections stained with Masson trichrome were used to quantify the content of ASM and the thickness of the epithelium. All airways cut transversally in 4 non-contiguous lung sections were analyzed, representing 7–25 airways per mouse (average of 17.0 ± 4.5). The content of airway smooth muscle in each airway was calculated by measuring the area occupied by the muscle divided by the square of its basement membrane perimeter [23]. The epithelium thickness was also analyzed on the same airways by measuring the area occupied by the epithelium divided by the basement membrane perimeter.

### Data Analysis

Individual data are presented, together with means ± standard deviations (SD). Two-way ANOVAs were used to assess the effect of pulmonary allergic inflammation, the mouse strain, and their interaction on each measured readout. When the interaction was significant, it was followed by a Sidak’s multiple comparisons test to specifically compare mice with and without pulmonary allergic inflammation in each mouse strain. All statistical analyses were performed with Prism (version 10.2.1, GraphPad, San Diego, CA). Differences with a *p *< 0.05 were considered statistically significant.

## Results

**Fig. 2:**
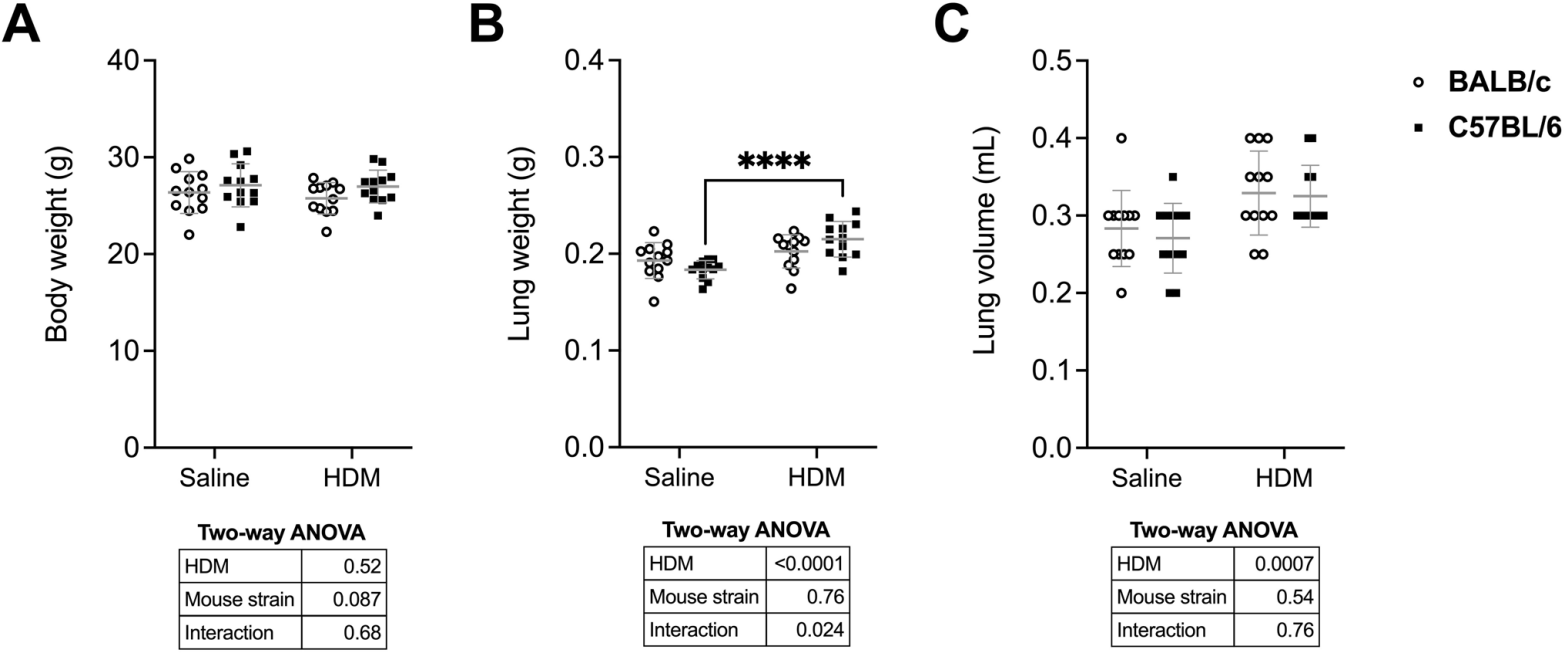
Body weight of mice (**A**)**,** total lung wet weight (**B**), and excised lung volume (**C**) are shown for BALB/c (open circles) and C57BL/6 (solid squares) mice. Data are individual results, together with means ± SD. Results of two-way ANOVAs are shown underneath the graphs. When the interaction was significant, a Sidak’s multiple comparison test was conducted and significant differences are indicated by asterisks (***p < 0.001). *N* = 12

The body weight was neither affected by HDM nor by the mouse strain (Fig. 2A). However, the weight and the volume of the excised lungs were elevated by HDM (Fig. 2B & C). There was also a significant interaction between HDM and the mouse strain for the weight of the lungs. Post hoc analysis indicated that this increase in lung weight caused by HDM was significant for the C57BL/6 mice but not for the BALB/c mice.

**Fig. 3:**
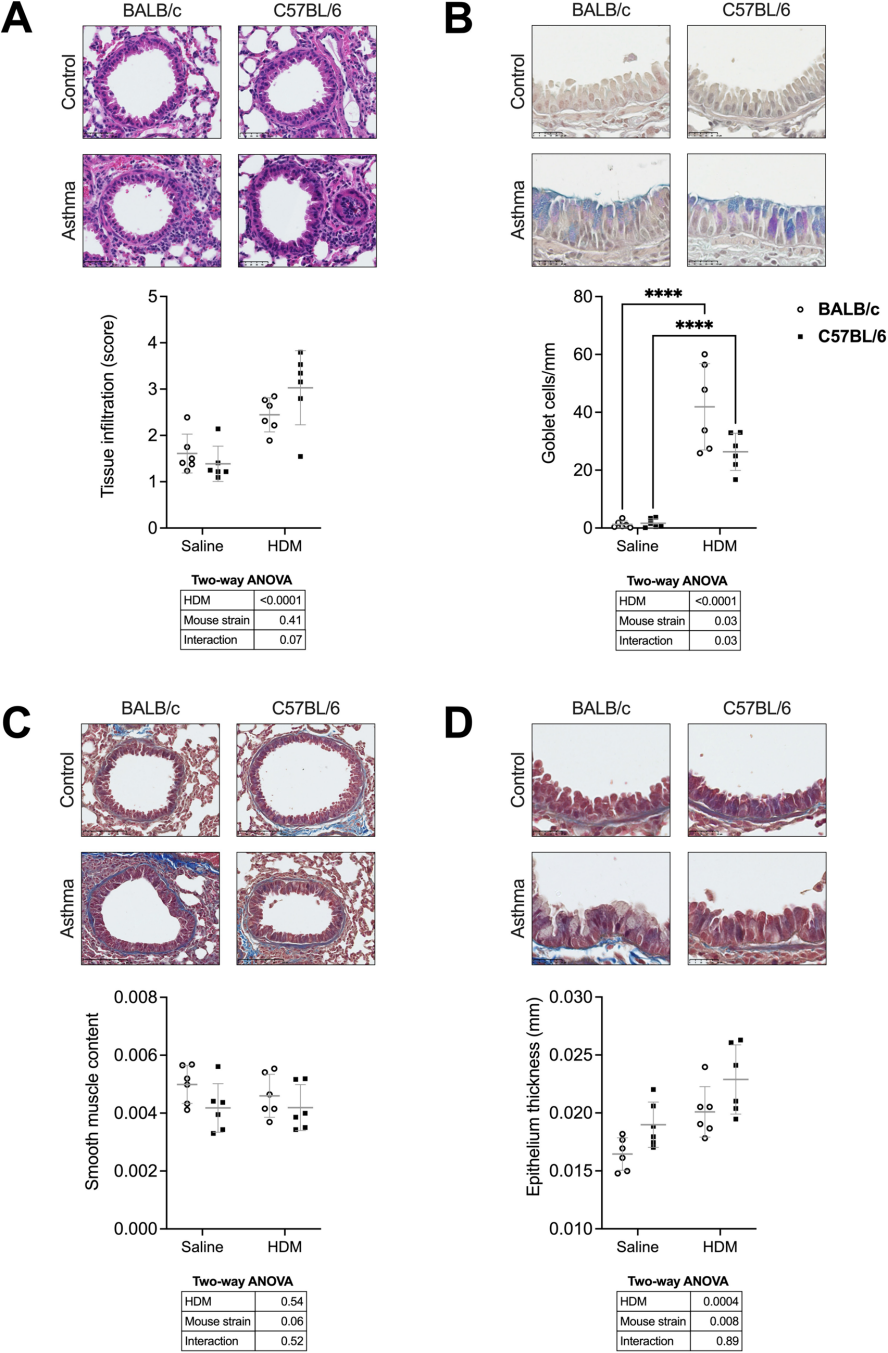
Histology score of tissue infiltration with inflammatory cells on lung sections stained with H&E (**A**), the number of goblet cells in the airway epithelium per mm of basement membrane on lung sections stained with PAS and alcian blue (**B**), and the content of smooth muscle per airway (**C**) and the epithelium thickness (**D**) on lung sections stained with Masson trichome are shown for BALB/c (open circles) and C57BL/6 (solid squares) mice. Representative histological images are shown on top, individual results with means ± SD are shown in scatterplots in the middle, and results of two-way ANOVAs are shown underneath the scatterplots. When the interaction was significant, a Sidak’s multiple comparisons test was conducted and significant differences are indicated by asterisks (****p < 0.0001). *N* = 6

The establishment of pulmonary allergic inflammation was confirmed by histology. The results are depicted in Fig. 3. Lungs of mice exposed to HDM exhibited a greater tissue infiltration with inflammatory cells (Fig. 3A), a greater number of goblet cells (Fig. 3B), no change in the content of airway smooth muscle (Fig. 3C), and an increased thickness of the airway epithelium (Fig. 3D). The histological alterations induced by HDM occurred to the same extent between the two mouse strains, except for the increased in goblet cells (interaction, *p* = 0.03), which was more pronounced in BALB/c mice. The epithelium was also thicker in C57BL/6 *versus* BALB/c mice (Fig. 3D).

**Fig. 4:**
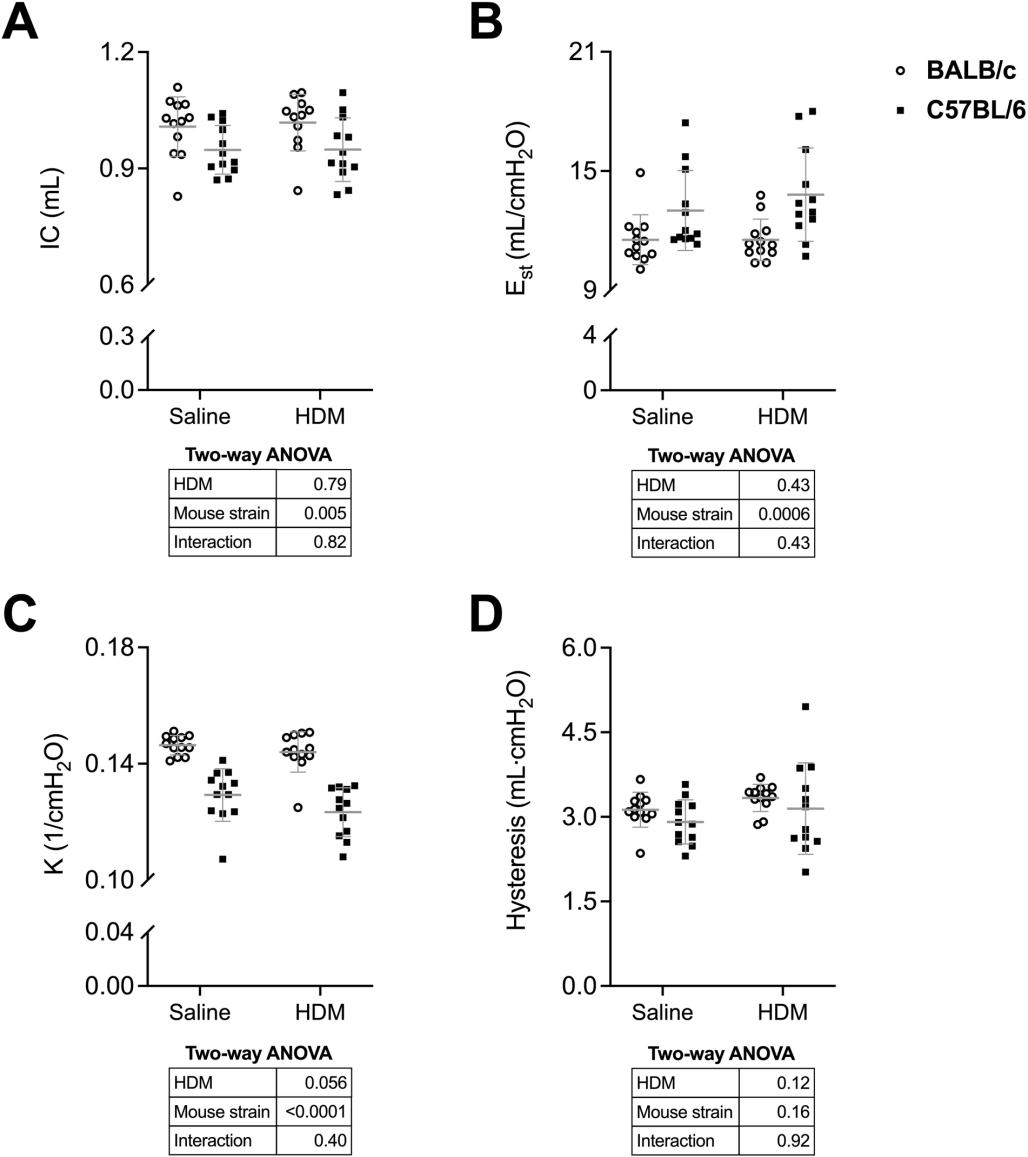
FlexiVent readouts from the deep inflation (DI) maneuver and the quasi-static, pressure-controlled partial pressure–volume (*P*–*V*) maneuver. Inspiratory capacity (*IC*) (**A**), quasi-static elastance (*E*_st_) (**B**), the parameter K of Salazar–Knowles equation (**C**), and hysteresis (**D**) are shown for BALB/c (open circles) and C57BL/6 (solid squares) mice. Data are individual results, together with means ± SD. Results of two-way ANOVAs are shown underneath the graphs. *N* = 12

Readouts from the deep inflation and the partial *P*–*V* maneuver are depicted in Fig. 4. None of these readouts were affected by HDM. As expected, the mouse strain significantly affected many of these readouts [14]. More precisely, *E*_st_ was greater, and IC and K were lower in C57BL/6 than BALB/c mice.

**Fig. 5:**
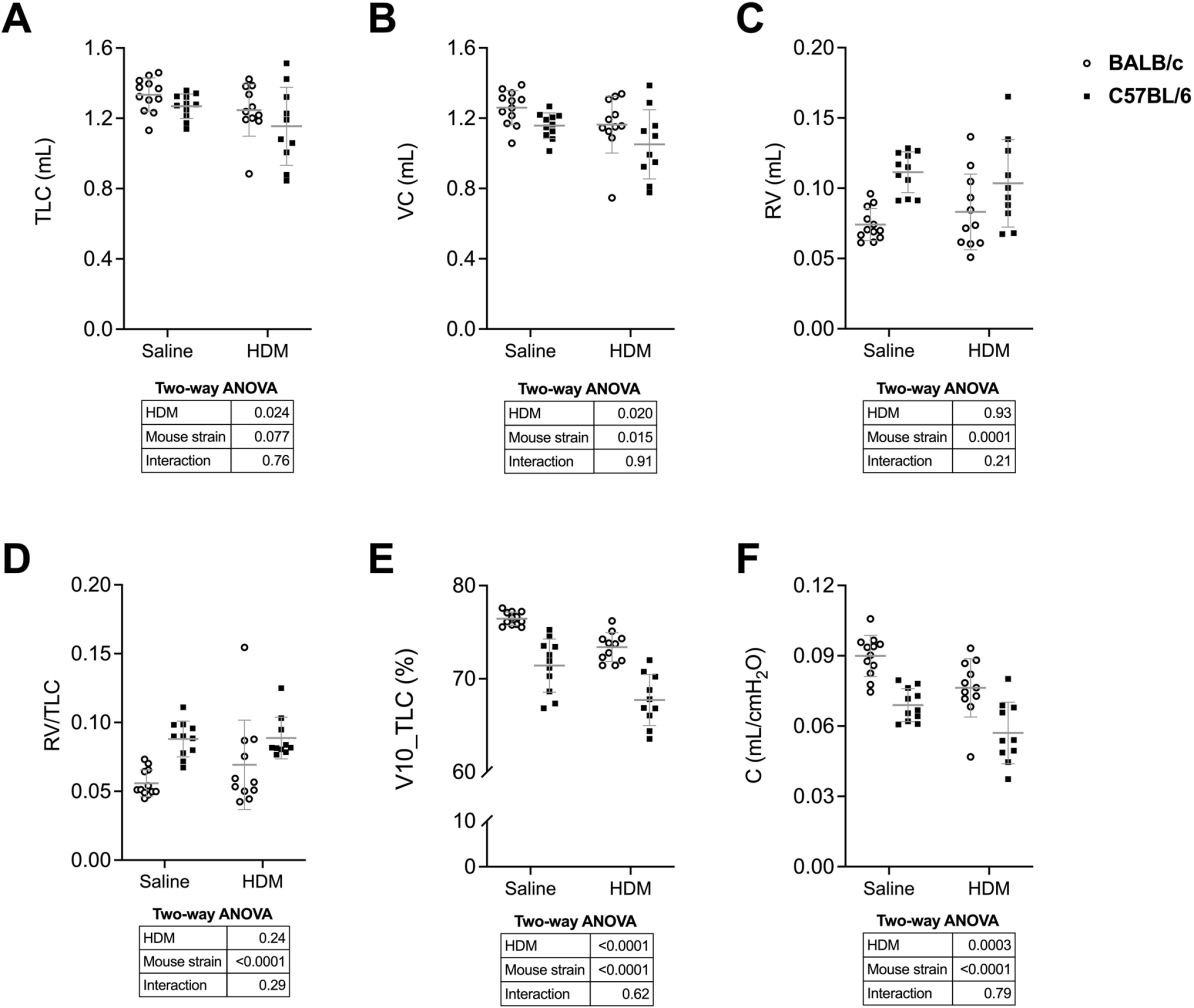
FlexiVent readouts from the dynamic, ramp-style, full-range pressure–volume (*P*–*V*) maneuver. Total lung capacity (TLC) (**A**), vital capacity (VC) (**B**), residual volume (RV) (**C**), RV/TLC (**D**), lung volume at 10-cm H_2_O expressed in percentage of TLC (V10_TLC) (**E**), and lung compliance (C) (**F**) are shown for BALB/c (open circles) and C57BL/6 (solid squares) mice. Data are individual results, together with means ± SD. Results of two-way ANOVAs are shown underneath the graphs. *N* = 10 to 12

Readouts from the full-range *P*–*V* maneuver are depicted in Fig. 5. The success rate for the full-range *P*–*V* maneuver was 92%, missing on one C57BL/6 mouse exposed to saline, one BALB/c mouse exposed to HDM, and two C57BL/6 mice exposed to HDM. The failed mice were detected by a volume in the deflation limb that was greater than in the inflating limb, indicating either an unsuccessful degassing or a flow leak. Four readouts were significantly decreased by HDM, namely TLC, VC, V10_TLC, and C. There were no effects of HDM on RV and RV/TLC. The alterations caused by HDM were also similar between the two mouse strains. As expected, most of these readouts were affected by the mouse strain [14]. More precisely, VC, V10_TLC, and C were lower and RV and RV/TLC were greater in C57BL/6 than BALB/c mice.

## Discussion

This study examines lung volumes in a mouse model of asthma. The establishment of pulmonary allergic inflammation was confirmed by histology, as well as by an increased size and volume of the excised lungs. While readouts from the deep inflation and the partial *P*–*V* maneuver were unable to detect a significant effect of HDM, many readouts from the full-range *P*–*V* maneuver were significantly different between mice with and without pulmonary allergic inflammation. These alterations, however, were inconsistent with the ones typically observed in human asthma.

Assessing lung volumes in mice is challenging. The automated method developed by Robichaud and co-workers [6] was meant to democratize the assessment of lung volumes in mice. The method stemmed from the original work of Soutiere and Mitzner [7] and Limjunyawong [8, 9], where the authors demonstrated that the method is successful for detecting alterations of lung volumes in mouse models of emphysema and fibrosis. Even though altered lung volumes are frequently observed in human asthma [3–5], the method had heretofore never been tested in a mouse model of asthma.

Overall, the results indicated that RV and RV/TLC were not significantly different between mice with and without pulmonary allergic inflammation. However, TLC and VC were significantly lower in mice exposed to HDM. The lung was also stiffer in mice exposed to HDM, attested by decreases in V10_TLC and C (Fig. 5). Interestingly, all these alterations were similar between mouse strains, suggesting that these responses to HDM are not specific to one mouse strain, but likely to be common in all mice. The only striking difference between C57BL/6 and BALB/c mice was a greater stiffness in the former, testified by an increase in *E*_st_ and decreases in K, V10_TLC, and C. The origin of this striking difference was recently described [14].

The apparent discrepancy between mice and humans in terms of lung volume alterations needs to be interpreted with caution. As aforementioned in the Introduction, lung volumes in humans are typically measured by plethysmography, and elevated RV and RV/TLC can then be appropriately interpreted as signs of air trapping. The method whereby RV and RV/TLC were measured in the present study is very different. During the 5 min of ventilation with 100% oxygen, a deep inflation is intercalated every 1 min to ascertain that all the trapped air behind closed airways is recruited and replaced by oxygen. During the subsequent 5 min of apnea, all oxygen, including the one behind closed airways, is thus absorbed. Air trapping is therefore intentionally removed before the full-range *P*–*V* maneuver. In the absence of air trapping, it is then not very surprising that both RV and RV/TLC are not affected by pulmonary allergic inflammation. It is argued that, because of the very different nature of the measurement, the changes in RV and RV/TLC in humans and mice need to be interpreted differently.

Different body postures may have also accounted for the apparent difference in lung volumes between mice and humans. While most data on humans are collected when the subjects are in an upright position, mice in the present study were studied in a supine position. According to our knowledge, lung volumes in mice have never been studied in an upright position. In humans, the lung was shown to behave like a Slinky, a spring distorting under its own weight [24]. This means that its tissue density progressively increases from the upper to the lower parts. The lower parts are thus more prone to airway closure and air trapping. In fact, closing volume increases with age [25, 26] because lung compliance progressively increases with age [27], like a loose Slinky with the bulk of coils dangling low. This tissue density gradient is also observed when recumbent [28]. However, because the weight of the lung is then distributed over a greater hanging surface, it decreases the effect of gravity and effectively decreases lung volumes [29, 30], including the dead space [31], fostering airway closure [30]. The latter phenomenon is thought to contribute to the greater occurrence of asthma symptoms at night [32, 33]. The tissue density gradient is also greater in the supine versus the prone position [28], probably explaining the smaller ventilation defects in asthma patients when prone [34]. Interestingly, mice also have a greater end-expiratory lung volume when prone compared to supine [35], suggesting that their lungs also behave like a Slinky. Taken together, these studies suggest that different body postures are unlikely to explain the lack of effect of HDM on RV and RV/TLC in the present study. This is because both recumbency (vs. upright) and supine (vs. prone) promote airway closure and air trapping in humans. The supine position should have thus amplified the effect of pulmonary allergic inflammation on lung volumes. Additionally, the increased RV in human asthma is still observed in supine position [29, 36]. Therefore, assuming that the lung response to postural changes is similar between mice and humans, an effect of pulmonary allergic inflammation on RV should have been seen in supine mice. The lack of effect is more likely due to technical differences in measurement between species as outlined above. In fact, using a plethysmographic method analogous to the one used in humans [37–39], RV was shown to be elevated in some mouse models of asthma [37]. Combining this latter method with the one used herein would be ideal for specifically quantifying air trapping in mice.

Another discrepancy with human asthma was decreased in TLC and VC, together with decreases in V10_TLC and C. In fact, TLC is sometimes increased in asthma [5] and, although not observed in all patients, an increased compliance has also been reported in some asthmatics [10, 11]. Another intriguing observation in the present study was that while lung volumes and compliance were affected when measured from the full-range *P*–*V* maneuver, the equivalent readouts were not affected when measured from the deep inflation or the partial *P*–*V* maneuver. Indeed, IC measured from the deep inflation and *E*_st_ measured from the partial *P*–*V* maneuver were not different between mice with or without pulmonary allergic inflammation. The only sign of stiffer lungs in HDM-exposed mice based on readouts from the partial *P*–*V* maneuver was a tendency toward a decreased K (*p* = 0.056). So why lung volumes and compliance are affected by HDM when measured from the full-range *P*–*V* maneuver but not when the lungs are probed with volume perturbations of smaller amplitudes?

Although the results are quite clear regarding this question, the interpretation is uncertain. It is tempting to speculate that edema, inflammatory infiltrates, and mucus accumulation ‘steal’ some of the volume that is normally available for air. Importantly, TLC measured herein is the amount of air that got into the mouse’s lungs and not the actual lung volume. Assuming no change in the volume of the structural component of the lung tissue, which is likely given the acute nature (10 days) of our mouse model, it would be expected that an inflammatory engorgement (i.e., combining interstitial tissue edema and/or accumulation of alveolar fluid) would reduce the amount of air that can get into the lungs. This would explain the decreased TLC and VC. The fact that the excised lung weight and volume were greater in mice with pulmonary allergic inflammation supports this idea. This inflammatory engorgement would also ‘steal’ the low lung volume; *i.e.*, the lower part of the *P*–*V* curve that is inherently more compliant. The rest of the lungs (*i.e.*, the part remaining for air) would thus be stiffer because it consists of the upper part of the *P*–*V* loop that is inherently less compliant. This would explain the lower compliance (V10_TLC and C). In other words, we think that the structural component of the tissue (i.e., not the fluid part) can be stretched up to a certain physical limit. And this physical limit would be the same irrespective of whether the stretch is induced by air accumulating in the alveoli during an inflation maneuver or by the fluid that built up within it (or around it) during the 10 days of HDM exposure.

We think that the changes in volumes and compliance cannot be perceived during smaller volume perturbations because the volume of the lungs at a PEEP of 3 cm H_2_O is presumably smaller in mice exposed to HDM compared to mice exposed to saline; perhaps as a result of a greater lung impedance due to small airway closure. The amount of air getting into the lungs from this PEEP all the way to 40-cm H_2_O (during the partial *P*–*V* maneuver) may therefore be the same as it would start at a lower lung volume in mice with pulmonary allergic inflammation. This would give the false impression that IC is not affected by HDM. It may also artificially mask the increased elastance (*i.e.*, E_st_) because: 1- the volume at which it is measured (i.e., 5 cm H_2_O during the deflation limb) may then be lower, increasing the apparent lung compliance and/or 2- the recruited air, which was not measured by the flexiVent during the inflation limb, then comes out of the lung during the deflation, artificially increasing the compliance by increasing the volume of air coming out per unit of pressure. More studies will be needed.

## Conclusion

Using a recently automated method with the flexiVent [6], this study demonstrated that while RV and RV/TLC are unaffected by pulmonary allergic inflammation, TLC and VC are decreased. Even though these alterations should be interpreted differently between mice and humans, we still argue that quantifying the extent by which these readouts are altered would be useful in pre-clinical studies to appraise the severity of experimental asthma, as well as to test the effectiveness of drugs in preventing or reversing experimental asthma.

## Data Availability

The datasets used and analyzed during the current study are available from the corresponding author on reasonable request.

## References

[1] Criee CP, Sorichter S, Smith HJ, Kardos P, Merget R, Heise D, Berdel D, Kohler D, Magnussen H, Marek W, Mitfessel H, Rasche K, Rolke M, Worth H, Jorres RA (2011) Working group for body plethysmography of the german society for P, and respiratory C. body plethysmography: its principles and clinical use. Respir Med 105:959–971 21356587 10.1016/j.rmed.2011.02.006

[2] Bhakta NR, McGowan A, Ramsey KA, Borg B, Kivastik J, Knight SL, Sylvester K, Burgos F, Swenson ER, McCarthy K, Cooper BG, Garcia-Rio F, Skloot G, McCormack M, Mottram C, Irvin CG, Steenbruggen I, Coates AL, Kaminsky DA (2023) European Respiratory Society/American Thoracic Society technical statement: standardisation of the measurement of lung volumes. Eur Respir J. 10.1183/13993003.01519-2022 37500112 10.1183/13993003.01519-2022

[3] Perez T, Chanez P, Dusser D, Devillier P (2013) Small airway impairment in moderate to severe asthmatics without significant proximal airway obstruction. Respir Med 107:1667–1674 24025779 10.1016/j.rmed.2013.08.009

[4] Tiwari A, Rahman K, Abejie B, Jain VV, Vempilly JJ (2017) Longer duration of asthma is significantly associated with increased RV/TLC ratio. Respir Med 124:44–48 28284320 10.1016/j.rmed.2017.01.011

[5] Kelly VJ, Brown NJ, Sands SA, Borg BM, King GG, Thompson BR (2012) Effect of airway smooth muscle tone on airway distensibility measured by the forced oscillation technique in adults with asthma. J Appl Physiol 112:1494–1503 22362406 10.1152/japplphysiol.01259.2011

[6] Robichaud A, Fereydoonzad L, Limjunyawong N, Rabold R, Allard B, Benedetti A, Martin JG, Mitzner W (1985) Automated full-range pressure-volume curves in mice and rats. J Appl Physiol 123(746–756):2017 10.1152/japplphysiol.00856.2016 PMC5668446 28751375

[7] Soutiere SE, Mitzner W (1985) On defining total lung capacity in the mouse. J Appl Physiol 96(1658–1664):2004 10.1152/japplphysiol.01098.2003 15075308

[8] Limjunyawong N, Craig JM, Lagasse HA, Scott AL, Mitzner W (2015) Experimental progressive emphysema in BALB/cJ mice as a model for chronic alveolar destruction in humans. Am J Physiol Lung Cell Mol Physiol 309:L662-676 26232300 10.1152/ajplung.00214.2015 PMC4593839

[9] Limjunyawong N, Fallica J, Horton MR, Mitzner W (2015) Measurement of the pressure-volume curve in mouse lungs. J Vis Exp. 10.3791/52376 25651276 10.3791/52376 PMC4354562

[10] Tonga KO, Berend N, Thamrin C, Farah CS, Jetmalani K, Chapman DG, King GG (2019) Lung elastic recoil and ventilation heterogeneity of diffusion-dependent airways in older people with asthma and fixed airflow obstruction. Eur Respir J. 10.1183/13993003.01028-2018 30578400 10.1183/13993003.01028-2018

[11] Tonga KO, Chapman DG, Farah CS, Oliver BG, Zimmermann SC, Milne S, Sanai F, Jetmalani K, Berend N, Thamrin C, King GG (2020) Reduced lung elastic recoil and fixed airflow obstruction in asthma. Respirology 25:613–619 31482693 10.1111/resp.13688

[12] Vincent NJ, Knudson R, Leith DE, Macklem PT, Mead J (1970) Factors influencing pulmonary resistance. J Appl Physiol 29:236–243 5428900 10.1152/jappl.1970.29.2.236

[13] Molfino NA, Slutsky AS, Julia-Serda G, Hoffstein V, Szalai JP, Chapman KR, Rebuck AS, Zamel N (1993) Assessment of airway tone in asthma comparison between double lung transplant patients and healthy subjects. Am Rev Respir Dis 148(1238):1243 10.1164/ajrccm/148.5.1238 8239160

[14] Rojas-Ruiz A, Boucher M, Gill R, Gelinas L, Tom FQ, Fereydoonzad L, Graham P, Soliz J, Bosse Y (2023) Lung stiffness of C57BL/6 versus BALB/c mice. Sci Rep 13:17481 37838793 10.1038/s41598-023-44797-x PMC10576825

[15] Boucher M, Henry C, Dufour-Mailhot A, Khadangi F, Bossé Y (2021) Smooth muscle hypocontractility and airway normoresponsiveness in a mouse model of pulmonary allergic inflammation. Front Physiol 12:698019 34267677 10.3389/fphys.2021.698019 PMC8277197

[16] Boucher M, Henry C, Khadangi F, Dufour-Mailhot A, Bossé Y (2021) Double-chamber plethysmography versus oscillometry to detect baseline airflow obstruction in a model of asthma in two mouse strains. Exp Lung Res 47:390–401 34541979 10.1080/01902148.2021.1979693

[17] Gill R, Rojas-Ruiz A, Boucher M, Henry C, Bossé Y (2023) More airway smooth muscle in males versus females in a mouse model of asthma: a blessing in disguise? Exp Physiol 108:1080–1091 37341687 10.1113/EP091236 PMC10988431

[18] Zosky GR, Janosi TZ, Adamicza A, Bozanich EM, Cannizzaro V, Larcombe AN, Turner DJ, Sly PD, Hantos Z (1985) The bimodal quasi-static and dynamic elastance of the murine lung. J Appl Physiol 105(685–692):2008 10.1152/japplphysiol.90328.2008 18556435

[19] Khadangi F, Tremblay-Pitre S, Dufour-Mailhot A, Rojas-Ruiz A, Boucher M, Henry C, Fereydoonzad L, Brunet D, Robichaud A, Bossé Y (2022) Sensitive physiological readouts to evaluate countermeasures for lipopolysaccharide-induced lung alterations in mice. Am J Physiol Lung Cell Mol Physiol 323:L107–L120 35670484 10.1152/ajplung.00073.2022

[20] Salazar E, Knowles JH (1964) An analysis of pressure-volume characteristics of the lungs. J Appl Physiol 19:97–104 14104296 10.1152/jappl.1964.19.1.97

[21] Boucher M, Henry C, Khadangi F, Dufour-Mailhot A, Tremblay-Pitre S, Fereydoonzad L, Brunet D, Robichaud A, Bossé Y (2022) Effects of airway smooth muscle contraction and inflammation on lung tissue compliance. Am J Physiol Lung Cell Mol Physiol 322:L294–L304 34936511 10.1152/ajplung.00384.2021

[22] Robichaud A, Fereydoonzad L, Limjunyawong N, Rabold R, Allard B, Benedetti A, Martin JG, Mitzner W (2017) Automated full-range pressure-volume curves in mice and rats. J Appl Physiol 123:746–756 28751375 10.1152/japplphysiol.00856.2016 PMC5668446

[23] Bullone M, Chevigny M, Allano M, Martin JG, Lavoie JP (1985) Technical and physiological determinants of airway smooth muscle mass in endobronchial biopsy samples of asthmatic horses. J Appl Physiol 117(806–815):2014 10.1152/japplphysiol.00468.2014 25103978

[24] Hopkins SR, Henderson AC, Levin DL, Yamada K, Arai T, Buxton RB, Prisk GK (1985) Vertical gradients in regional lung density and perfusion in the supine human lung: the slinky effect. J Appl Physiol 103(240–248):2007 10.1152/japplphysiol.01289.2006 PMC2399899 17395757

[25] Anthonisen NR, Danson J, Robertson PC, Ross WR (1969) Airway closure as a function of age. Respir Physiol 8:58–65 5366416 10.1016/0034-5687(69)90044-9

[26] King GG, Eberl S, Salome CM, Young IH, Woolcock AJ (1998) Differences in airway closure between normal and asthmatic subjects measured with single-photon emission computed tomography and technegas. Am J Respir Crit Care Med 158:1900–1906 9847284 10.1164/ajrccm.158.6.9608027

[27] Colebatch HJ, Greaves IA, Ng CK (1979) Exponential analysis of elastic recoil and aging in healthy males and females. J Appl physiol Respir Environ Exerc Physiol 47:683–691 511674 10.1152/jappl.1979.47.4.683

[28] Tawhai MH, Nash MP, Lin CL, Hoffman EA (2009) Supine and prone differences in regional lung density and pleural pressure gradients in the human lung with constant shape. J Appl Physiol 107:912–920 19589959 10.1152/japplphysiol.00324.2009 PMC2755995

[29] Duggan CJ, Watson RA, Pride NB (2004) Postural changes in nasal and pulmonary resistance in subjects with asthma. J Asthma 41:701–707 15584628 10.1081/jas-200027820

[30] Clague HW, Hall DR (1979) Effect of posture on lung volume: airway closure and gas exchange in hemidiaphragmatic paralysis. Thorax 34:523–526 505349 10.1136/thx.34.4.523 PMC471109

[31] Fowler WS (1950) Lung function studies. Iv. postural changes in respiratory dead space and functional residual capacity. J Clin Invest 29:1437–1438 16695814 10.1172/JCI102382 PMC436189

[32] Gustafsson PM (2003) Pulmonary gas trapping increases in asthmatic children and adolescents in the supine position. Pediatr Pulmonol 36:34–42 12772221 10.1002/ppul.10310

[33] Ballard RD, Pak J, White DP (1991) Influence of posture and sustained loss of lung volume on pulmonary function in awake asthmatic subjects. Am Rev Respir Dis 144:499–503 1892286 10.1164/ajrccm/144.3_Pt_1.499

[34] Harris RS, Winkler T, Musch G, Vidal Melo MF, Schroeder T, Tgavalekos N, Venegas JG (2009) The prone position results in smaller ventilation defects during bronchoconstriction in asthma. J Appl Physiol 107:266–274 19443742 10.1152/japplphysiol.91386.2008 PMC2711796

[35] Takahashi T, Sakai N, Nishino S (2021) Altered responses of end-expiratory lung volume and upper airway patency to body posture in diet-induced obese mice. Physiol Rep 9:e15072 34676689 10.14814/phy2.15072 PMC8531836

[36] Ballard RD, Irvin CG, Martin RJ, Pak J, Pandey R, White DP (1990) Influence of sleep on lung volume in asthmatic patients and normal subjects. J Appl Physiol 68:2034–2041 2361905 10.1152/jappl.1990.68.5.2034

[37] Vanoirbeek JA, Rinaldi M, De Vooght V, Haenen S, Bobic S, Gayan-Ramirez G, Hoet PH, Verbeken E, Decramer M, Nemery B, Janssens W (2010) Noninvasive and invasive pulmonary function in mouse models of obstructive and restrictive respiratory diseases. Am J Respir Cell Mol Biol 42:96–104 19346316 10.1165/rcmb.2008-0487OC

[38] Janosi TZ, Adamicza A, Zosky GR, Asztalos T, Sly PD, Hantos Z (2006) Plethysmographic estimation of thoracic gas volume in apneic mice. J Appl Physiol 101:454–459 16645196 10.1152/japplphysiol.00011.2006

[39] Larcombe AN, Zosky GR, Bozanich EM, Turner DJ, Hantos Z, Sly PD (2008) Absence of cholinergic airway tone in normal BALB/c mice. Respir Physiol Neurobiol 161:223–229 18440286 10.1016/j.resp.2008.01.009

